# Mapping of a Novel Quantitative Trait Locus Conferring Bacterial Blight Resistance in the Indigenous Upland Rice Variety ULR207 Using the QTL–Seq Approach

**DOI:** 10.3390/plants14142113

**Published:** 2025-07-09

**Authors:** Tanawat Wongsa, Sompong Chankaew, Tidarat Monkham, Meechai Siangliw, Niranjan Baisakh, Jirawat Sanitchon

**Affiliations:** 1 Department of Agronomy, Faculty of Agriculture, Khon Kaen University, Khon Kaen 40002, Thailand; t.wongsa@kkumail.com (T.W.); somchan@kku.ac.th (S.C.); tidamo@kku.ac.th (T.M.); 2 National Center for Genetic Engineering and Biotechnology (BIOTEC), National Science and Technology, Development Agency (NSTDA), Khlong Luang, Pathum Thani 12120, Thailand; meechai@biotec.or.th; 3 School of Plant, Environmental and Soil Sciences, Louisiana State University Agricultural Center, Baton Rouge, LA 70803, USA; nbaisakh@agcenter.lsu.edu

**Keywords:** KASP marker, SNP index, R gene, bulk segregant analysis, rice germplasm

## Abstract

Bacterial blight (BB) disease is a serious stress that affects up to 80% of rice yield. Utilizing an elite resistant variety was previously thought to be an alternative way to control disease outbreaks. The indigenous upland rice variety ULR207 is a high-potential donor for the BB resistance breeding program. However, the quantitative trait loci (QTLs) associated with bacterial blight resistance in this variety have not yet been discovered. Therefore, QTLs associated with BB resistance need to be identified. In this study, we identified the QTLs associated with BB resistance in the F_2:3_ population crossed between the BB resistance variety ULR207 and Maled Phai, as well as a susceptible variety, via QTL-seq analysis and bulk-segregant analysis. We found a new QTL-associated BB resistance locus (*qBBchr8*) mapped on chromosome 8. Five positions were candidates, including *Os08g0110700*, *Os08g0115200*, *Os08g0131300*, *Os08g0139500*, and *Os08g0163900*. Afterwards, Kompetitive Allele-Specific PCR (KASP) markers specific to the SNP variant and the position of each gene were designed. These markers, associated with the disease lesion length phenotype, were validated with another 178 individual plants of the F_2_ population via single-marker analysis. This analysis revealed that the position *Os08g0110700* was the strongest locus, with a PVE of 15.00%. The results suggest that this KASP SNP marker could be used to improve elite rice for BB resistance.

## 1. Introduction

Rice (*Oryza sativa* L.) is a staple cereal crop consumed as a carbohydrate source by more than 50% of the people worldwide, and 90% of the total rice in the world is produced by Asian countries [1,2]. However, rice production and yield are affected by epidemic bacterial blight (BB) disease. BB, which is caused by *Xanthomonas oryzae* pv. *oryzae* (*Xoo*), is a severe and destructive rice disease worldwide which causes a yield loss of more than 50% under *Xoo*-preference climates [3]. Chemical application is an effective means to control the disease, but, it is neither environmentally friendly nor cost-effective. Thus, developing BB-resistant varieties is the most appropriate and economical approach to controlling the disease [4,5].

Several BB resistant varieties have been reported, such as WC1263, IRBB5, Minh-Soc, Ganga Sagar, IRBB21, P8, IR73571-3B-11-3-K3, *O. glaberrima* IRGC102600B, and Mutant H120, and all these varieties carry a single R gene [6,7,8,9,10,11]. However, these BB-resistant varieties carrying a single R gene could lose their resistance ability due to pathogen adaptation [12]. Breeding rice varieties with multiple R genes is a better approach to coping with this problem [13,14,15]. Breeding lines have been developed for broad-spectrum resistance through pyramiding R genes in different combinations, such as *Xa4* + *xa5* + *Xa21*, *xa5* + *xa13* + *Xa21*, *xa5* + *Xa21*, *Xa21* + *Xa33*, and *Xa23* with other genes, and *Xa4* + *xa5* + *Xa7* + *xa13* + *Xa21*, providing more effective resistance to *Xoo* than individual resistance genes [16,17,18,19,20,21,22]. Identifying new sources of bacterial blight resistance is urgently needed for breeding varieties with durable resistance.

More than 40 genes associated with BB resistance have been reported so far [23,24]. Some of these resistance genes have been transferred from rice germplasms to develop rice cultivars with durable resistance by gene pyramiding [25,26]. Despite numerous resistance genes being identified and used for breeding, there are many resistance genes that remain unexplored and have not been used as resistance sources [5]. Five indigenous lowland rice varieties in Thailand were found to be resistant against *Xoo* and have preferable agronomic traits [27]. Also, Chumpol et al. [28] reported Thai indigenous upland rice varieties with bacterial blight resistance to Thai *Xoo* isolates. The results showed that the indigenous upland rice variety ULR207 exhibits strong broad-spectrum BB resistance and possesses a non-analogous gene with resistant check varieties [29], suggesting that this might be a novel gene. However, the gene associated with BB resistance in this variety has not been discovered. Thus, further study to identify QTLs associated with resistance is needed.

The QTL-seq method is known to be a rapid way to identify the QTLs related to traits of interest in plants [30]. A mapping population is constructed based on a target phenotype of two varieties, which show the extreme phenotype. Then, two DNA bulks from the individual progenies with the extreme phenotype (e.g., resistant and susceptible pools) in the population are sequenced [30]. Bulk segregant analysis (BSA) with whole-genome sequencing can show differences in the patterns of single-nucleotide polymorphism (SNP) indices between two bulk populations, which can be used to identify the QTLs in each chromosome [20]. Numerous QTLs have been reported in rice using the QTL-seq method for traits such as seed vigor and blast resistance [5,30], cold tolerance in wild rice (*Oryza rufipogon*) [31], grain length and weight [32], low phosphorus tolerance [33], and grain elongation [34]. In this study, we endeavored to employ QTL-seq together with BSA for the identification of BB resistance QTLs in the indigenous upland rice variety ULR207, which will facilitate molecular rice breeding for bacterial blight resistance.

## 2. Results

### 2.1. Evaluation of BB Resistance and Bulk Population Construction

**Figure 1:**
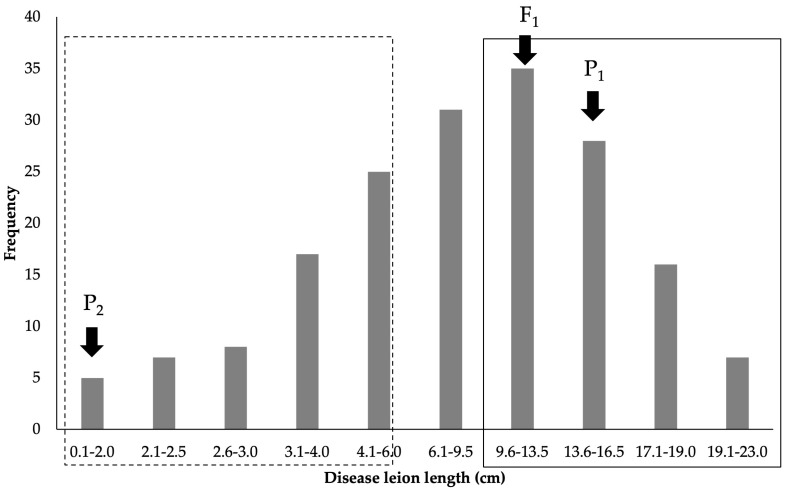
The frequency distribution of BB disease reaction in the F_2_ population and two parental lines, obtained via the leaf clipping method. BRB: BB-resistant bulk (dotted-line rectangle); BSB: BB-susceptible bulk (solid-line rectangle).

The F_2_ mapping population and parental lines were evaluated for BB resistance at the seedling stage via a clipping method against SP1-1 isolate. The result showed that the recipient parent, Maled Phai (P_1_), was disease-susceptible, with an average lesion length of 15.82 ± 2.30 cm, while the donor parent, ULR207 (P_2_), was resistant, with an average lesion length of 1.21 ± 0.82 cm. This indicates that the susceptible variety Maled Phai and resistant variety ULR207 exhibited extremely contrasting reactions, while the F_1_ population was susceptible, with an average lesion length of 13.30 ± 3.16 cm, demonstrating recessive control of BB resistance. In the F_2_ population, the disease lesion length ranged between 0.1 and 21.5 cm. The resistance bulk had a range of disease lesion lengths from 0.1 to 5.0 cm, while susceptible bulk had a range from 12.6 to 21.5 cm (Figure 1). The resistant and susceptible bulks showed contrasting disease reactions, thus validating their suitability for QTL-seq analysis.

### 2.2. Whole Genome Resequencing and Read Mapping

DNA libraries of BB-resistant bulk, BB-susceptible bulk, and the two parental lines were used for whole-genome resequencing on an Illumina HiSeq. 2500 platform. Approximately 97 million reads each for the BRB, BSB, and donor parent (ULR207) and 92 million reads for the recurrent parent (Maled Phai) were generated, yielding 16.4 Gb, 16.3 Gb, 16.5 Gb, and 15.8 Gb for the BRB, BSB, donor parent, and recipient parent, respectively. The average sequencing depths of the BRB, BSB, recipient parent, and donor parent were 36.46, 36.16, 36.93, and 34.89, respectively. The alignment of the reads from both the BRB and BSB, along with the parents, to the reference genome of Nipponbare was 98.47%, 98.35%, 95.99% and 96.48% for reads mapped in the BRB, BSB, ULR207, and Maled Phai, respectively, corresponding to 97.90%, 97.76%, 95.99%, and 96.48% of rice genome coverage (Table 1). The high-quality reads of Maled Phai were used to generate its reference sequence. Mapping against the reference sequence of Maled Phai was performed to identify the common SNPs in the BRB and BSB. A total of 740,253 SNPs were identified between the two parents, supported by at least 29 reads, of which 522,983 SNPs supported by at least 3 reads were obtained in two bulks, the BRB and BSB. The SNPs between the bulks were selected for further calculation of the SNP index and ∆(SNP index) (Table 2).

### 2.3. Candidate Genomic Region for BB Resistance

**Figure 2:**
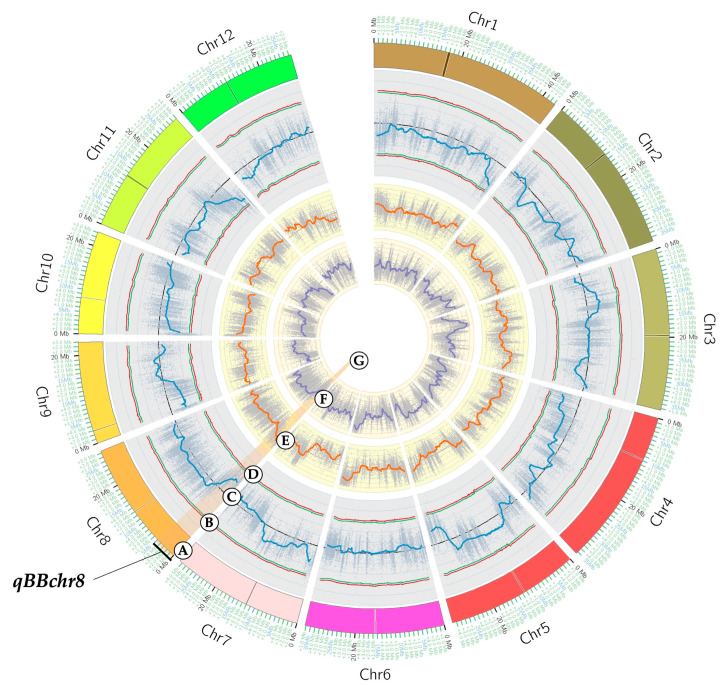
Circos plot of BRB and BSB based on SNP index plots obtained by subtracting between them. (**A**) Psuedomolecules of Nipponbare reference genome (IRGSP 1.0); (**B**,**D**) probability values at 95% (green line) and 99% confidence (red line); (**C**) average SNP index in each plot with 2 Mb window size and 10 kb increment; (**E**) SNP index plots of BRB with 2 Mb window size and 10 kb increment; (**F**) SNP index plots of BSB with 2 Mb window size and 10 kb increment; and (**G**) candidate genomic regions containing QTLs for BB resistance.

Based on the ∆SNP index plot, which was high and above the threshold (confidence intervals > 95%), a genomic region on chromosome 8, namely *qBBchr8*, was identified by QTL-seq as the candidate QTL associated with BB resistance in ULR207 (Figure 2). The results showed that the BRB mostly contained the ULR207 type genome at this QTL, while the BSB mostly had the Maled Phai type genome.

**Figure 3:**
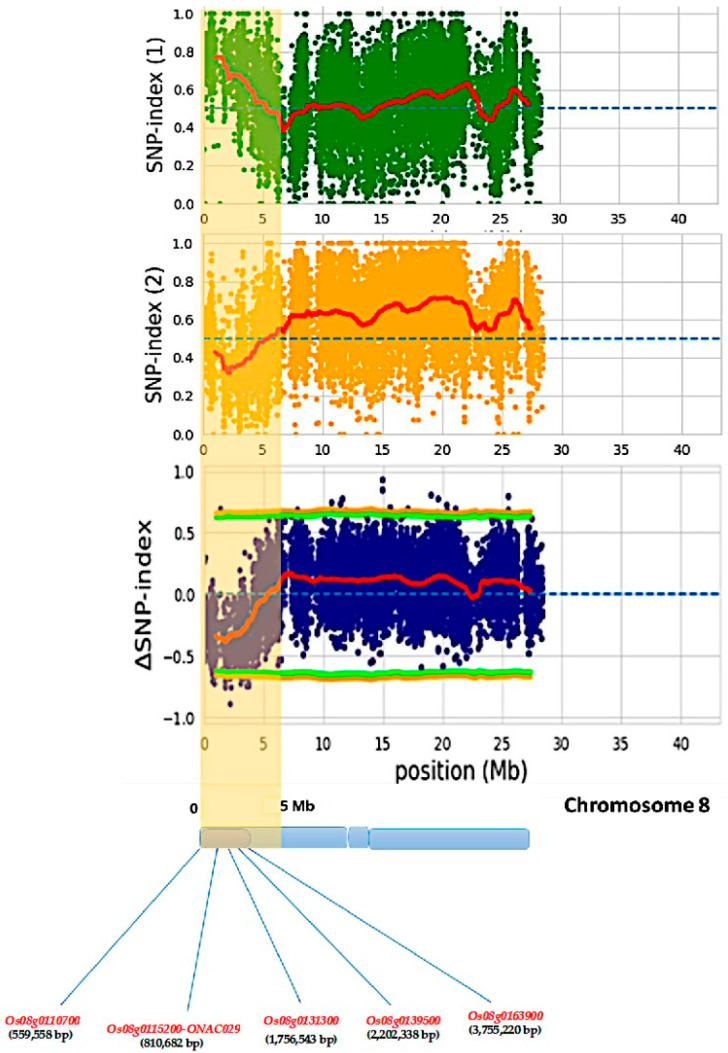
SNP index plots of the resistant bulk (top, BRB) and susceptible bulk (middle, BSB), and ∆(SNP index) plot (bottom) for chromosome 8. The light-yellow color highlights the detected QTL regions with distinguishing SNP indexes in the two bulks. The green and orange lines are the threshold, while the red line is the average of SNP index of the two bulks.

The *qtlBBchr8* was mapped to a 5.0 Mb interval (between 0 and 5.0 Mb) that contained 523 annotated genes. Of these, 339 genes in the 4 Mb region encompassing *qtlBBchr8* contained nonsynonymous SNPs. From these, only five genes (Figure 3)—*Os08g0110700*, encoding phosphatidic acid-preferring phospholipase A1; *Os08g0115200*, encoding microtubule-localized IQ-domain containing protein (OsNAC029); *Os08g0131300* (*GPAT* domain-containing protein); *Os08g0139500*, coding for a low-density lipoprotein receptor-related protein; and *Os08g0163900*, encoding a protein of unknown function, DUF569 domain-containing protein—that had high value of ∆(SNP index) were selected as the candidate genes in the *qBBchr8* region involved in the plant disease-resistance mechanism.

### 2.4. Validation and Confirmation of the Identified QTL on Chromosome 8

Primers for KASP assay were designed for the five candidate genes for BB disease resistance (*Os08g0110700*, *Os08g0115200*, *Os08g0131300*, *Os08g0139500*, and *Os08g0163900* (Figure 3), based on the sequences of each gene. The 178 F_2_ plants were genotyped using KASP assay, and validation of markers’ associations with disease lesion length was performed via single-marker analysis. The four markers, including *Os08g0110700*, *Os08g0131300*, *Os08g0139500*, and *Os08g0163900* showed an additive effect in the negative direction, indicating that the presence of these genes caused a decrease in disease lesion length, while *Os08g0115200* exhibited an additive effect in the opposite direction. Also, the five markers explained 1.93–15.00% of the phenotypic variance. The marker linked to *Os08g0110700* had 15.00% phenotypic variance explained (PVE), with an LOD score of 6.32; the *Os08g0115200* marker had 14.60% PVE, with an LOD score of 6.13; the *Os08g0163900* marker explained 4.37%, with an LOD score of 1.73; the *Os08g0131300* marker explained 2.77%, with an LOD score of 1.09; and the *Os08g0139500* marker explained 1.93%, with an LOD score of 0.75 (Table 3). The results demonstrate that both the *Os08g0110700* and *Os08g0115200* genes, with high PVE percentages, have a major effect on bacterial blight resistance, while other loci exhibit minor effects on the trait.

## 3. Discussion

QTL-seq has been used successfully to identify QTLs in various crops, such as rice [5,26,35], foxtail millet [36], squash [37], pigeon pea [38], cucumber [39], and groundnut [40]. This study identified five genes associated with disease lesion length linked to *qtlBBchr8* located on chromosome 8. These genes are located near chromosomal positions known for their role in defense against plant diseases. Validation of the KASP markers by single-marker analysis showed that *Os08g0110700* and *Os08g0115200* had a strong associations with BB resistance, with PVE values of 15.00% and 14.60%, respectively. These two are suggested as major genes for BB resistance in ULR207.

BB resistance genes in rice have been reported and classified into four groups: (1) the receptor-like kinase (RLK) gene (*Xa3*/*Xa26*, *Xa4*, and *Xa21*), involved in PAMP-Triggered Immunity (PTI) [41,42,43]; (2) the sugar and are eventually exported as a transporter (*SWEET*) gene (*Xa13*, *Xa25*, and *Xa41*); (3) executor proteins (*Xa10*, *Xa23*, and *Xa27*); and (4) other types (*Xa1* and *xa5*) [11]. In the present study, we identified *Os08g0110700* and *Os08g0115200* as significant genes with high PVE percentages. *Os08g0110700* was involved in encoding the phosphatidic acid pathway, which is related to leaf cell death in Arabidopsis [44] and could induce reactive oxygen species (ROS) generation in Arabidopsis [45], rice [46], tobacco, and potato [47]. ROS generation in response to pathogen infestation transduces signaling to plant defense mechanisms such as hypersensitive response (HR) [48]. HR is a form of rapid cell death at the point of pathogen infection induced by several pathogens, such as fungi, oomycetes, viruses, and bacteria [49]. BB resistance in rice has been reported to involve HR due to the activation of the salicylic acid (SA) metabolic pathway [50]. We hypothesize that the phosphatidic acid pathway might be involved in the HR-mediated disease defense mechanism in the indigenous rice variety ULR207. However, the other three genes, *Os08g0131300*, *Os08g00139500*, and *Os08g0163900*, have been identified as minor genes with low PVE percentages, and they also have another interpretation option, that is, that the minor effects of the three genes are caused by the linkage effect of the main locus *Os08g0110700*. Our results report a novel QTL (*qBBchr8*) on chromosome 8, which could benefit future rice BB resistance breeding programs.

KASP markers have been developed for QTLs identified by QTL-seq analysis, and have consequently been utilized for marker-assisted selection in numerous crops, such as blast disease resistance in rice [5], cooked-grain elongation in rice [34], bacterial blight resistance (gene *xa5*) and bacterial leaf streak resistance in rice (*Oryza sativa* L.) [51], *Phytophthora* crown rot resistance in squash [37], and rust and late-leaf spot resistance in groundnut (*Arachis hypogaea* L.) [40]. In this study, validation of the KASP marker for *Os08g0110700*, linked to the QTL on chromosome 8, showed an association with resistance to *Xoo* isolates. This promising marker could be utilized to support rice breeding programs by decreasing the time consumption of breeding through cost-effective marker-assisted selection.

## 4. Materials and Methods

### 4.1. Construction of Mapping Populations

**Figure 4:**
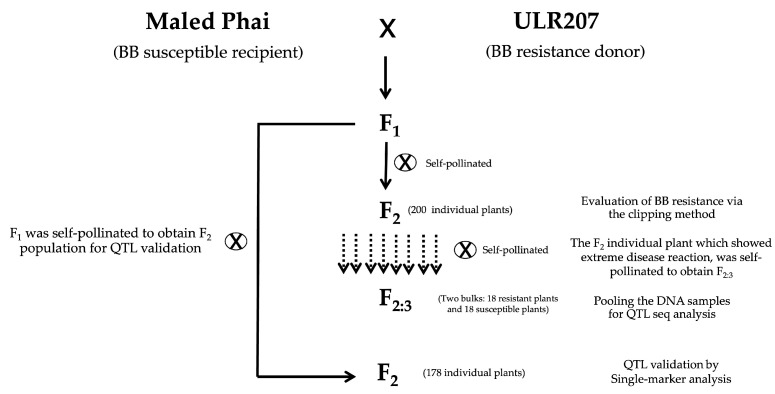
Construction of the mapping population from a cross between Maled Phai and ULR207.

The bi-parental mapping (F_2:3_) population was developed from the varieties Maled Phai (MP) and ULR207. The female parent, Maled Phai, is susceptible to bacterial blight disease, whereas the donor parent, ULR207, is resistant. These varieties were kindly provided by the Rice Project, Khon Kaen University, Khon Kaen, Thailand. To develop the mapping population, an F_1_ plant from the Maled Phai/ULR207 cross was self-pollinated to generate F_2_ seeds. Individual F_2_ plants (F_2_ individual line) were grown for BB resistance evaluation. These individual F_2_ plants, which were identified as resistant and susceptible lines, were then self-pollinated to obtain F_3_ seeds. F_3_ individual plants were grown to develop resistant and susceptible bulk for QTL-seq analysis (Figure 4).

### 4.2. Evaluation of Bacterial Blight Resistance

**Figure 5:**
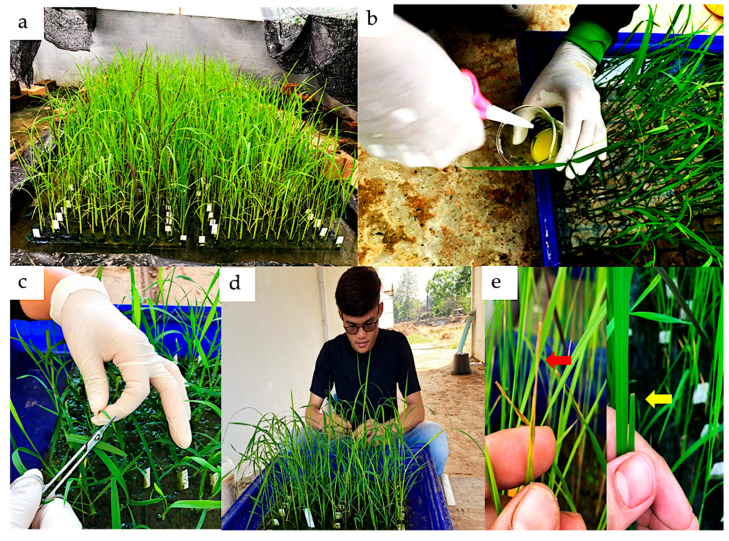
The overall process of *Xoo* inoculation and evaluation of BB disease resistance under the greenhouse conditions: (**a**) rice seedlings under the greenhouse conditions; (**b**) the sterile scissors dipped into the bacterial suspension; (**c**) inoculation of *Xoo* by the clipping method; (**d**) the measurement of disease lesion length at 10 DAI; (**e**) the BB disease symptoms of the susceptible (red arrow) and resistant varieties (yellow arrow).

Two hundred seeds of the F_2_ individual (lines), together with their parental lines, were evaluated for bacterial blight resistance against the SP1-1 *Xoo* isolate via the clipping method [52]. In the preliminary experiment, the SP1-1 isolate (Suphan buri 1-1) was chosen, due to it having a significantly different disease reaction from the parental line (Table S1) [29]; detailed information regarding the isolate’s characteristics is provided in the Supplementary Materials (Table S2). The seeds were soaked for two days, then grown in 72-hole trays inside a greenhouse maintained at a relative humidity of ~85–93% and a temperature of ~35 °C for 21 days after planting or until the 3rd leaf stage. The *Xoo* SP1-1 isolate was used to inoculate the plants. Twenty-one days after planting the seedlings, the leaf blade was cut at 2–3 cm from the leaf tip using sterile scissors and then dipped in the bacterial suspension, which was adjusted to a concentration of 10^9^ CFU/mL (OD = 0.3 at 600 nm) [53]. The disease lesion length of the individual plant was measured after 10 days of inoculation (DAI) (Figure 5). The classification of disease reaction was adapted from the standard evaluation system of IRRI [54]: a BB lesion length of less than 5 cm was classified as resistant, while a lesion length of more than 5 cm was identified as susceptible [55,56].

### 4.3. Construction of Bulks, DNA Extraction, and Whole-Genome Resequencing

Two-hundred individual F_2_ plants, obtained by crossing the BB-susceptible variety Maled Phai and the BB-resistant variety ULR207, were selected based on disease reaction data, and then the F_2:3_ population was generated. The individual plants of the F_2:3_ population were selected based on disease reaction, as mentioned above. The resistant and susceptible bulks consisted of 18 resistant individual F_2:3_ plants and 18 susceptible individual F_2:3_ plants, respectively. Young leaves of individual F_2:3_ plants were extracted to obtain DNA using the GeneJET Plant Genomic DNA Purification MiniKit (Thermo Scientific™, Waltham, MA, USA). The individual DNA samples from the 18 BB-resistant and 18 BB-susceptible plants were pooled to create resistant bulk (BRB) samples and susceptible bulk (BSB) samples, respectively. The two bulked DNA and two parental DNA samples were used to prepare whole-genome DNA-seq libraries, which were sequenced using the Illumina HiSeq 2500 platform (Illumina, Inc., Hayward, CA, USA).

### 4.4. QTL-Seq Analysis

The QTL–seq pipeline was used for QTL-seq analysis as described by Takagi [30]. To obtain high-quality reads, raw reads were trimmed for low-quality and adapter-containing sequences. The BWA aligner was used to align the clean reads of two bulk samples and the high-quality reads of Maled Phai to the reference genome of Nipponbare (IRGSP1.0) [57]. Afterward, substitution of the Nipponbare reference genome with the variants representing Maled Phai was used to generate a Maled Phai reference genome. The identification of DNA variants calling for SNPs (single nucleotide polymorphisms) and Indel (small insertion/deletion) in the BRB and BSB was performed by aligning the BAM files containing high-quality reads of both bulks onto the Maled Phai reference genome using Samtools [58]. SNP indices at each SNP position for the BRB and BSB were calculated following the methods described by Takagi et al. [30] and Abe et al. [59]. SNPs with an SNP index < 0.3 in both bulks were excluded, and the remaining SNPs (SNP index ≥ 0.3 in either bulk) were considered as the real SNPs. The ∆ SNP index was calculated by subtracting the SNP index of the BSB from that of the BRB. The ∆(SNP index) and average SNP index were estimated for a given genomic region using a sliding window size of 2 Mb with 10 kb increments to generate SNP index plots for all rice chromosomes. Plots comparing the average SNP index and ∆(SNP index) between the two bulks were visualized using Circos [60]. A QTL associated with BB resistance in ULR207 was determined as a peak or valley in the an SNP index plot which showed an average ∆(SNP index) higher than the statistical confidence intervals under the null hypothesis of no QTL (*p*-value < 0.05) [30].

### 4.5. Annotation of Candidate QTLs Associated with BB Resistance

The variance effect predictor (VEP: https://plants.ensembl.org/oryza_sativa/Tools/VEP) (accessed on 24 November 2024) was used to determine the effect of the SNPs in each locus. Loci with nonsynonymous SNPs and other SNPs called from the comparison of the resequencing data of the two parental lines Maled Phai and ULR207 that were related to plant disease defense mechanisms were selected as candidate genes [5,43].

### 4.6. Marker Development and Marker–Trait Association Analysis

The QTL on chromosome 8 related to the BB-resistant phenotype was validated in 178 individual F_2_ plants using five Kompetitive Allele-Specific PCR (KASP) markers developed for nonsynonymous SNPs in *Os08g013950* (T/C at 2,002,338 bp), *Os08g016390* (C/T at 3,755,220 bp), *Os08g0115200* (C/T at 810,658 bp), *Os08g0110700* (A/G at 559,558 bp), and *Os08g0131300* (A/T at 1,756,543 bp, Genotyping with KASP markers was performed using the method described earlier [43]) (Tables S3–S6). Single-marker analysis was performed using the KASP genotypic data and the phenotypic data of the 178 individual F_2_ lines. A simple regression analysis was performed to determine the phenotypic variance explained by each QTL [43].

## 5. Conclusions

This study successfully identified a new QTL (*qBBchr8*) on chromosome 8 associated with BB resistance in the indigenous upland rice variety ULR207. Five genes, *Os08g0110700*, *Os08g0115200*, *Os08g0131300*, *Os08g0139500*, and *Os08g0163900*, were considered as candidate genes. *Os08g0110700*, which was found to have high PVE for BB resistance, was considered to be the best candidate gene. The KASP marker developed for this gene could be helpful in further rice breeding programs.

## Figures and Tables

**Table 1:** Summary of Illumina sequencing data of resistant bulk, susceptible bulk, and parental lines.

Samples	Clean Reads	Clean Data (Gb)	Read Alignment (%)	Genome Coverage (%)	Average Depth
BRB	97,295,280	13,965,515,760	98.47%	97.90%	36.46
BSB	96,870,311	13,870,606,503	98.36%	97.76%	36.16
ULR207	98,220,506	14,103,458,885	98.66%	96.49%	36.93
MP	93,419,258	13,401,558,279	98.23%	95.99%	34.89

BRB = BB-resistant bulk, BSB = BB-susceptible bulk, MP = Maled Phai.

**Table 2:** Chromosome-wise distribution of single-nucleotide polymorphisms (SNPs) between the two bulks: BRB and BSB.

Chromosomes	Length (bp)	Number of SNPs
1	43,270,923	59,553
2	35,937,250	40,447
3	36,413,819	38,670
4	35,502,694	45,999
5	29,958,434	40,303
6	31,248,787	45,331
7	29,697,621	32,174
8	28,443,022	56,841
9	23,012,720	28,260
10	23,207,287	45,270
11	29,021,106	49,445
12	27,531,856	40,690
Total	373,245,519	522,983

**Table 3:** Single-marker analysis of five KASP markers associated with disease lesion length of F_2_ population.

Markers	Chromosome	LOD	PVE (%)	Additive Effect	Dominant Effect
*Os08g0110700*	8	6.32	15.00	−1.20	−0.20
*Os08g0115200*	8	6.13	14.60	1.10	−0.52
*Os08g0131300*	8	1.09	2.77	−0.48	−0.23
*Os08g0139500*	8	0.75	1.93	−0.45	−0.31
*Os08g0163900*	8	1.73	4.37	−0.66	0.01

LOD = logarithm of odds, and PVE = phenotypic variance explained.

## Data Availability

The data are contained within this article and are available upon request.

## Supplementary Materials

The following supporting information can be downloaded at https://www.mdpi.com/article/10.3390/plants14142113/s1: Table S1: Disease lesion length of 17 rice varieties at 17 DAI against 10 *Xoo* isolates under greenhouse conditions; Table S2: Isolation of the origin pathogen, genome sequencing information, and population structure of *Xanthomonas oryzae* pv. *oryzae* (*Xoo*) strain; Table S3: Information about KASP primer assay for QTL-seq validation; Table S4: Information about sequencing reads of P_1_, P_2_, resistant bulk, and susceptible bulk; Table S5: Alignment data of P_1_, P_2_, resistant bulk, and susceptible bulk; Table S6: Information of RAD gene annotation for P_1_, P_2_, resistant bulk, and susceptible bulk.
