# Supplementary material for: Mapping of a Novel Quantitative Trait Locus Conferring Bacterial Blight Resistance in the Indigenous Upland Rice Variety ULR207 Using the QTL–Seq Approach
Source: Plants (Basel). 2025 Jul 9;14(14):2113. doi: 10.3390/plants14142113 (PMC12298398; doi:10.3390/plants14142113)
Supplement: Supplementary file 1 [file plants-14-02113-s001.zip › Supplementary data 1(Tables S1 and S2).pdf]

Table S1. Disease lesion length of 17 rice varieties at 17 DAI against 10 *Xoo* isolates under greenhouse condition

Varieties	Isolates code / Disease lesion length (cm.)									
	UT2-1	CM4-1	CM3-1	NB7-7	PR5-1	NB7-8	CN2-1	NY1-1	SP1-1	MS1-2	BSR
ULR207	6.58	11	3.96	8.66	5.53	4.81	5.85	12.73	6.09	8.18	0.8
Maled Phai	13.31	13.79	11.02	15.18	12.72	11.87	11.18	12.56	17.51	17.71	0
RD6	17.28	19.92	16.88	21.34	16.27	19.64	12.01	20.69	21.34	22.33	0
KDML105	21.84	21.8	18.55	26.81	19.14	24.49	16.54	17.7	25.96	22.32	0
IRBB21	16.19	18.53	13.89	16.31	13.96	15.96	10.21	13.13	17.05	17.96	0
IRBB1	3.03	8.94	0.76	7.18	8.64	4.89	5.38	7.4	7.5	7.87	1.0
IRBB3	6.28	8.77	6.94	6.23	3.59	5.27	3.04	4.02	5.58	6.17	1.0
IRBB14	15.25	16.26	12.65	15.22	13.98	19.14	10.88	15.97	14.92	16.99	0
IRRBB13	18.36	15.78	12.74	16.64	16.01	12.98	10.82	15.13	15.98	16.37	0
IR21	18.35	16.74	13.46	19.07	14.48	17.63	9.77	15.99	12.72	20.87	0.1
IRBB11	13.41	9.88	11.09	15.73	12.94	16.03	9.72	11.11	12.32	13.43	0.2
IRBB4	15.64	4.87	7.71	16.52	13.93	17.97	10.89	5.82	12.48	18.04	0.3
IRBB5	4.08	1.61	1.79	2.19	1.89	2.46	2.14	3.31	1.11	1.63	1.0
IR62266	13.86	2.38	2.03	9.32	12.88	10.18	8.32	1.77	11.64	12.02	0.7
IRBB8	8.98	6.15	7.39	7.88	6.15	5.09	4.53	10.45	7.12	7.26	1.0
IRBB10	16.17	14.07	13.19	12.98	13.3	18.75	9.67	15.21	16.84	18.75	0
IRBB7	1.85	0.9	0.28	2.33	2.31	0.61	0.47	0.26	0.14	2.07	1.0
F-test	**	**	**	**	**	**	**	**	**	**	
C.V. %	18.52	17.61	12.41	16.75	23.41	18.38	19.68	39.73	23.75	20.72	

** Significant at p = 0.01, BSR = Broad=spectrum resistance

Table S2. Isolation of the origin pathogen, genome sequencing information, and population structure of *Xanthomonas oryzae* pv. *oryzae* (*Xoo*) strain

Strains	District	Province	Collection year	Infected rice varieties	%GC content	Total length (bp)	Genes number	CDSs number	%Mapping	SNP	Indel	Total SNP and Indel	Cluster of population structure	Clade of PCA and NJ
SP1-1	Si Prachan	Suphan Buri	2551	Phitsanulok 2	64.01	4,245,577	3,808	3,753	98.50	14,370	984	15,354	2	1
